# Synthesis of ZnAl_2_O_4_ and Evaluation of the Response in Propane Atmospheres of Pellets and Thick Films Manufactured with Powders of the Oxide

**DOI:** 10.3390/s21072362

**Published:** 2021-03-29

**Authors:** Emilio Huízar-Padilla, Héctor Guillén-Bonilla, Alex Guillén-Bonilla, Verónica-María Rodríguez-Betancourtt, A. Sánchez-Martínez, José Trinidad Guillen-Bonilla, Lorenzo Gildo-Ortiz, Juan Reyes-Gómez

**Affiliations:** 1 Facultad de Ciencias Químicas, Universidad de Colima, Colima 28400, Colima, Mexico; ehuizar@ucol.mx; 2 Departamento de Ingeniería de Proyectos, CUCEI, Universidad de Guadalajara, M. García Barragán 1421, Guadalajara 44410, Jalisco, Mexico; 3 Departamento de Ciencias Computacionales e Ingenierías, CUVALLES, Universidad de Guadalajara, Carretera Guadalajara-Ameca Km 45.5, Ameca 46600, Jalisco, Mexico; alex.guillen@academicos.udg.mx; 4 Departamento de Química, CUCEI, Universidad de Guadalajara, M. García Barragán 1421, Guadalajara 44410, Jalisco, Mexico; veronica.rbetancourtt@academicos.udg.mx; 5 CONACYT-Unidad Académica de Ciencias Químicas, Universidad Autónoma de Zacatecas, Campus Siglo XXI, Carretera Zacatecas—Guadalajara Km 6, Ejido la Escondida, Zacatecas 98160, Zacatecas, Mexico; asanchezma@conacyt.mx; 6 Departamento de Electrónica, CUCEI, Universidad de Guadalajara, M. García Barragán 1421, Guadalajara 44410, Jalisco, Mexico; trinidad.guillen@academicos.udg.mx; 7 Departamento de Física, CUCEI, Universidad de Guadalajara, Guadalajara 44410, Jalisco, Mexico; lorenzo.gildo@academicos.udg.mx; 8 Facultad de Ciencias, Universidad de Colima, Bernal Díaz del Castillo 340, Colima 28045, Colima, Mexico; reyesgj@ucol.mx

**Keywords:** ZnAl_2_O_4_, nanoparticles, dynamic response, gas sensors

## Abstract

ZnAl_2_O_4_ nanoparticles were synthesized employing a colloidal method. The oxide powders were obtained at 300 °C, and their crystalline phase was corroborated by X-ray diffraction. The composition and chemical structure of the ZnAl_2_O_4_ was carried out by X-ray and photoelectron spectroscopy (XPS). The optical properties were studied by UV-vis spectroscopy, confirming that the ZnAl_2_O_4_ nanoparticles had a direct transition with bandgap energy of 3.2 eV. The oxide’s microstructures were microbars of ~18.2 nm in size (on average), as analyzed by scanning (SEM) and transmission (TEM) electron microscopies. Dynamic and stationary gas detection tests were performed in controlled propane atmospheres, obtaining variations concerning the concentration of the test gas and the operating temperature. The optimum temperatures for detecting propane concentrations were 200 and 300 °C. In the static test results, the ZnAl_2_O_4_ showed increases in propane response since changes in the material’s electrical conductance were recorded (conductance = 1/electrical resistance, Ω). The increases were ~2.8 at 200 °C and ~7.8 at 300 °C. The yield shown by the ZnAl_2_O_4_ nanoparticles for detecting propane concentrations was optimal compared to other similar oxides categorized as potential gas sensors.

## 1. Introduction

For decades, the preparation processes of inorganic materials have had great relevance for obtaining optimal microstructures for potential technological applications. Traditionally, the solid-state reaction technique (or ceramic method) has been the most commonly used to synthesize different types of materials [1]. However, some research groups have reported alternative routes of synthesis, such as wet chemistry, which is simple, economical, easy to apply, and can be very efficient for obtaining high purity inorganic materials at low temperatures [2]. Other synthesis routes are the sol–gel, coprecipitation, ultrasonic spray, and colloidal methods [3,4,5,6]. Recently, it has been reported that the colloidal method is a very effective preparation route, with which it is possible to obtain different types of microstructures [2]. For example, reference [7] reports that by the colloidal method, it is possible to obtain different morphologies by applying the optimal synthesis conditions. In reference [8], microspheres and nanoparticles of CoSb_2_O_6_ are synthesized and successfully proved as gas sensors. The wet chemistry processes have allowed reducing the particle size to a nanometric scale, thus favoring the increase of both the surface area and the physical and chemical properties of the material [9]. The nanometric size of the particles increases their electrical response, in addition to the improvement of their magnetic, optical, and catalytic properties [10,11]. All this implies that materials with nanometric particle size can potentially be applied as gas sensors [2].

Tin oxide (SnO_2_) and zinc oxide (ZnO) nanoparticles are the most commonly used materials as gas sensors [12]. However, semiconductor oxides with more complex crystalline structures have also been suggested as alternative materials to be applied as gas detectors, like LaCoO_3_ [13], ZnSb_2_O_6_ [14], CoAl_2_O_4_ [15], CoNb_2_O_6_ [16], and more recently ZnAl_2_O_4_ [17]. The high response shown by these semiconductors in atmospheres of CO, CO_2_, LPG, ethanol, and in humid environments is mainly due to the type of morphology, the high porosity and the nanometer size of the particles. It is important to mention that the cited works do not investigate the detection of propane gas, although reference [5] reports the detection of CO and propane using LaCoO_3_ nanoparticles.

In the case of the oxide ZnAl_2_O_4_ (with a bandgap of 3.5–3.9 eV [18]), its nanoparticles show interesting physical and chemical properties that make it suitable for different technological applications, mainly in the areas of solid-state lighting and displays, catalysis, ultraviolet (UV) photoelectronic devices, thermal control coatings for spacecraft, transparent conductors, optical coating devices, mechano-optical stress sensors, and microwave dielectric devices [19], among others. The oxide can be represented by the stoichiometric formula XAl_2_O_4_, wherein X=Co, Ni, Cu, or Zn [20]. The material generally crystallizes into a spinel-type structure with space group Fd3m [18] and has normally been prepared by the traditional method of synthesis [21], although very effective wet chemistry processes have been reported for obtaining nanoparticles of this compound [6,22].

In this work, ZnAl_2_O_4_ nanoparticles were synthesized using the microwave-assisted colloidal method, and their potential use as a gas sensor was studied. After thorough literature research, we found that the ZnAl_2_O_4_ has already been applied as a sensor for humidity and other gases. However, no evidence was found that this oxide has been previously used as a propane sensor. For this reason, we studied the dynamic and static response of ZnAl_2_O_4_ nanoparticles in propane atmospheres, obtaining excellent results. A high purity crystalline phase of the oxide was obtained at 300 °C. Gas detection experiments using pellets made with ZnAl_2_O_4_ powders showed high sensitivity when the operating temperatures and the C_3_H_8_ gas concentrations were varied.

## 2. Experimental

### 2.1. Sample Preparation

Spinel-type ZnAl_2_O_4_ was synthesized by a non-aqueous method assisted by microwave radiation. For that purpose, three 5 mL solutions of absolute ethyl alcohol (CTR) were prepared as a solvent for 0.01 mol of Al(NO_3_)_3_·9H_2_O (Sigma-Aldrich, Jalmek, Guadalajara, Mexico), 0.005 mol of Zn(NO_3_)_2_·6H_2_O (Jalmek, Guadalajara, Mexico), and 0.5 mL of ethylenediamine (Sigma, Guadalajara, Mexico). The solutions were stirred at 375 rpm for 20 min. Subsequently, the three solutions were mixed slowly to obtain a colloidal suspension, which was kept under constant stirring for 24 h at room temperature. After that, the solvent was evaporated by placing the suspension in a conventional domestic microwave oven (LG, model MS1147 X, Guadalajara, Mexico), applying a power of 140 W. The solution was irradiated 40 times, lasting 60 s each irradiation. The energy absorbed by the solution was estimated at 336 kJ. To avoid splashing and the consequent loss of material, the solution was kept at around 70 °C, measuring the temperature with an Extech 403,255 infrared thermometer. The purpose of using microwave radiation in the synthesis process is to elucidate its effects on ZnAl_2_O_4_’s microstructure. After evaporation, the obtained paste was dried in air at 200 °C for 8 h, and the precursor material was afterward calcined at 300 °C. The calcination was done in a Novatech programmable oven with temperature control (Tlaquepaque, Mexico), starting at room temperature and reaching 300 °C at a heating rate of 100 °C/h for 5 h.

### 2.2. Physical Characterization of ZnAl_2_O_4_ Powders

Purity and crystallinity of the ZnAl_2_O_4_ powders were characterized by X-ray diffraction at room temperature using a Panalytical Empyrean device with CuKα radiation (λ = 1.546 Å). The diffraction was done using a 2θ continuous scan from 10° to 80° with 0.026°-steps at a rate of 30 s per step. The powders’ microstructure calcined at 300 °C was analyzed by means of a field-emission scanning electron microscope (FE-SEM, Tescan MIRA 3 LMU, Mexico City, Mexico) with an acceleration voltage of 10 kV in a high vacuum. The shape and size of the nanoparticles were studied with a transmission electron microscope (TEM, Joel JEM-ARM200F, Mexico City, Mexico) in image mode. For this, a representative amount (0.010 g) of the powders was placed in a vial containing methanol. Subsequently, the powders and the methanol were dispersed by an ultrasonic generator for 10 min and deposited on a 300-mesh copper grid containing a Formvar/carbon membrane.

### 2.3. Chemical and Optical Characterization of the ZnAl_2_O_4_

Chemical composition and surface analysis of the ZnAl_2_O_4_ calcined at 300 °C were studied using an X-ray photoelectron spectroscopy system (XPS SPECS, Berlin, Germany) equipped with a monochromatic Al Kα_1_ (hv = 1486.7 eV) X-ray source and a hemispherical energy analyzer (PHOIBOS 150). To determine the value of the forbidden bandwidth and the material’s percentage of reflection, a Cary 5000 UV-vis NIR spectrophotometer (Agilent Technologies, Santa Clara, United States) was used, equipped with a polytetrafluoroethylene (PTFE) integration sphere.

### 2.4. Pellets and Thick Films Preparation for Gas Sensing Tests

For the detection tests in propane atmospheres, pellets and thick films were made from the powders calcined at 300 °C. For the pellets, 0.350 g of ZnAl_2_O_4_ powders were compressed using a manual hydraulic press (Simplex Ital Equip-25 tons, Mexico City, Mexico), applying a 10-ton load for 1 min. The dimensions of the pellets were 12 mm in diameter and 0.5 mm in thickness. For the thick films, 0.1 g of ZnAl_2_O_4_ powders was used, which was dispersed with an ultrasonic generator (Branson 2510 Ultrasonic) in a plastic container containing ethyl alcohol. The dispersed powders were then dropwise placed in a ceramic ring so that there was a contact between the radially separated electrodes at two tips (“two-tips method”). The dimensions of the thick films were 0.3 mm in diameter and 0.5 mm in thickness.

**Figure 1:**
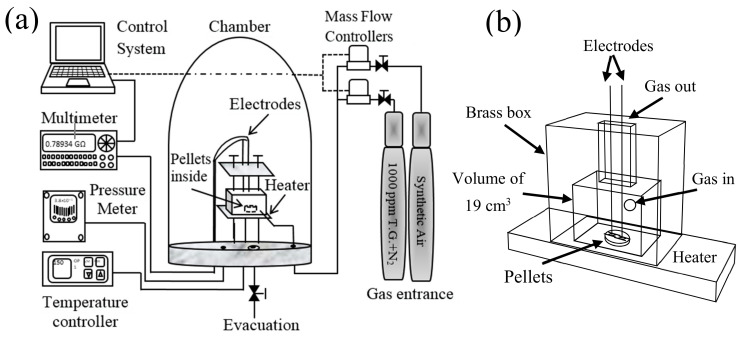
(**a**) Schematic of the system used to perform dynamic and static tests in direct current (DC) on pellets of the oxide at different concentrations and operating temperatures, and (**b**) Box used for the input and output of our propane gas.

During the sensing tests in static and dynamic propane atmospheres, changes in the material’s electrical resistance (in DC) were expected depending on the propane concentration, the operating temperature, and the testing time (dynamic response). For that, two ohmic contacts were placed on the pellets’ surface using colloidal silver paint (Alfa Aesar, 99%, Mexico City, Mexico). Subsequently, the pellets were placed in a small box (V = 19 cm^3^) placed inside the measurement chamber (see Figure 1a). The purpose of this small box was to ensure fast response times. The box had two holes: one through, which the electrodes were inserted, and which also functioned as a gas outlet; the other orifice worked as the gas inlet (see Figure 1b). The gas was then removed through the evacuation system installed in the vacuum chamber (which had a pressure of 10^−3^ torr). In the case of the static tests (as a function of the concentration and temperature), the total volume (10 L) of the vacuum chamber was used. The partial pressure of the test gases was monitored by an electronic detector (Leybold TM20, Oerlikon Leybold Vacuum, Cologne, Germany). The change in electrical resistance was measured with a digital multimeter (Keithley 2001, Cleveland, OH, USA) coupled to a control and acquisition system using the software LabView (National Instruments). For controlling the propane flow during the dynamic tests, a pair of massflow regulators (2600 and 10 cm^3^/min) were used (Brooks Instruments, GF100CXXC-SH452.6 L and GF100CXXC-SH40010C), at a total flow rate of 500 cm^3^/min. To estimate the static response (S) as a function of concentration and operating temperature, the relative difference of the electrical conductances (1/electrical resistance, Ω) was considered according to the formula S = (G_G_ − G_O_)/G_O_ [5], where G_G_ and G_O_ are the pellets’ electrical conductance in propane and air, respectively.

For the dynamic tests with the thick films, these were placed in a quartz tube inside a tubular-type oven with programmable temperature control (Lindberg Blue M). The measurements were made at 200 and 300 °C by the two-tips method employing high purity platinum wires with a diameter of 0.006 mm. The control of the gas concentrations was made in the same manner as in the pellet detection tests.

## 3. Results and Discussion

### 3.1. XRD Analysis

**Figure 2:**
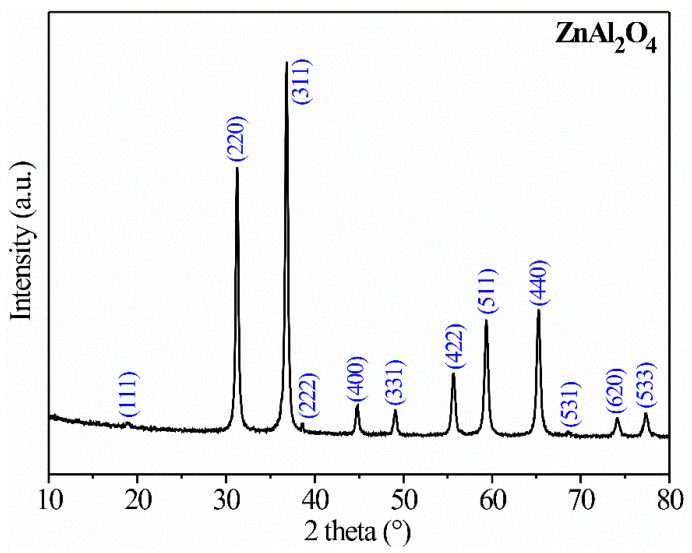
X-ray diffractograms of ZnAl_2_O_4_ powders calcined at 300 °C.

Figure 2 shows an X-ray diffractogram of powders calcined at 300 °C. The diffraction peaks were located at 2θ angular positions: 18.9°, 31.2°, 36.8°, 38.5°, 44.8°, 49.07°, 55.6°, 59.3°, 32.6°, 68.6°, 74.1°, and 77.3° corresponding to the crystalline planes (111), (220), (311), (222), (400), (331), (422), (511), (440), (531), (620), and (533), respectively.

The ZnAl_2_O_4_′s cell parameter was calculated using the equation [23,24]:

(1) $$a=\;\frac{\lambda \sqrt{\left(h^{2}+\;k^{2}+\;l^{2}\right)}}{2\sin\theta },$$

where *a* is the lattice constant, *λ* is the wavelength of the radiation (1.5406 Å), (*h*,*k*,*l*) are the Miller indices, and *θ* is the Bragg angle. Considering all the reflections, the estimated lattice parameter for the ZnAl_2_O_4_ was 8.060 Å with a standard deviation of 0.007 Å [24]. This result is consistent with those reported in the literature for the same compound [18,20,21,24]. The material’s crystalline phase was identified by means of the PDF file 05–0669 corresponding to the cubic phase of the ZnAl_2_O_4_, with a spinel-like structure and an Fd-3m (227) spatial group [22].

The synthesis method employed in this work was successful for obtaining the spinel-like crystalline structure of the ZnAl_2_O_4_ at a relatively low-temperature (300 °C) compared to other works where the same oxide was synthesized but using other preparation methods. For example, zinc aluminate has been synthesized via a coprecipitation approach followed by a heat treatment at 900 °C [17,18]. ZnAl_2_O_4_ powders were obtained by heating a precursor in the air at 900 °C (pyrolysis) [20]. MAl_2_O_4_ (M = Ni, Cu, Zn) spinels have been prepared by the sol–gel autocombustion method in the temperature range of 1000–1200 °C [22]. In the present study, the use of ethylenediamine contributed to the formation of the crystalline phase at relatively low temperatures. This amine is a ligand that can bind to metal ions forming coordination compounds. When the ethylenediamine reacts with a transition-metal nitrate, this produces a coordinated complex whose conformation can act as a directing agent of structure [5].

### 3.2. Analysis of Chemical and Optical Properties

**Figure 3:**
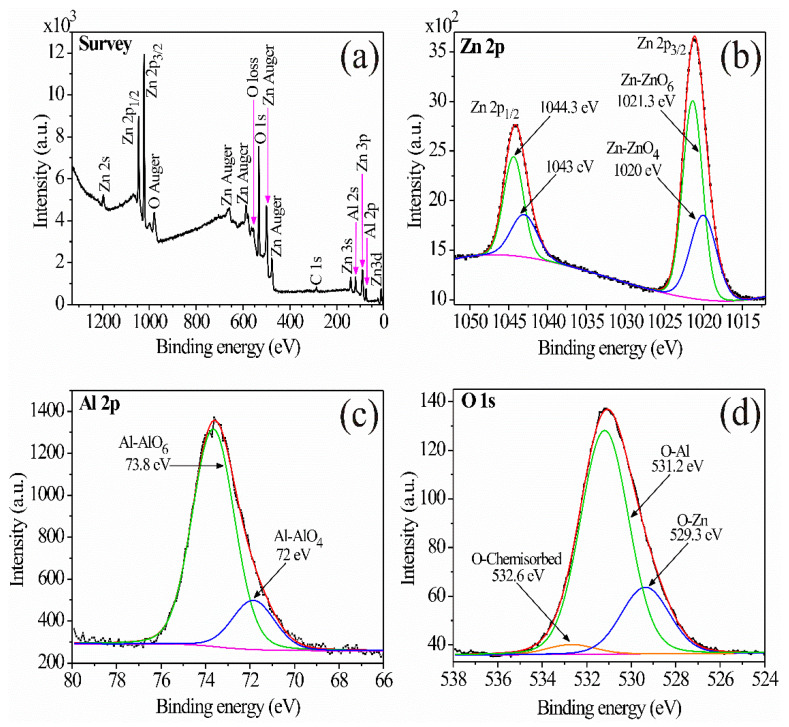
XPS-spectra: (**a**) survey, (**b**) Zn 2p, (**c**) Al 2p, (**d**) O1s of ZnAl_2_O_4_.

To determine the chemical composition of the calcined oxide at 300 °C, the XPS technique was applied. Figure 3a shows the elements on the material’s surface. All spectra taken from the deep levels corresponding to Al 2p, Zn 2p, and O 1s were aligned with respect to the adventitious carbon C 1s centered at 284.8 eV and deconvolved with the software AAnalyzer (RDATAA, Mexico City, Mexico) [25]. The XPS spectrum associated with the Zn 2p (Figure 3b) was deconvolved considering two doublets: The first one at 1020 and 1043 eV associated with Zn^2+^ ions located at the tetrahedral sites of the ZnAl_2_O_4_ spinel network; the second one at 1021.3 and 1044.3 eV associated with Zn^2+^ ions located at the octahedral sites in the spinel network [26,27]. Figure 3c shows the XPS spectrum associated with Al 2p, which was deconvolved using two doublets located around 72 and 73.8 eV at the tetrahedral and octahedral sites, respectively, and associated with Al^3+^ ions. The XPS spectrum associated with O 1 s (Figure 3d) was deconvolved using three singlets located at 529.3, 531.2, and 532.6 eV. The 529.3 and 531.2 peaks were associated with the O^2−^ ions on the O-Zn and O-Al bonds, so it can be assumed that these ions are located at the ZnO_x_ and AlO_x_ sites (tetrahedral or octahedral) in the spinel [28]. The peak located at 532.6 eV was associated with the chemisorbed oxygen on the material’s surface [29,30].

**Figure 4:**
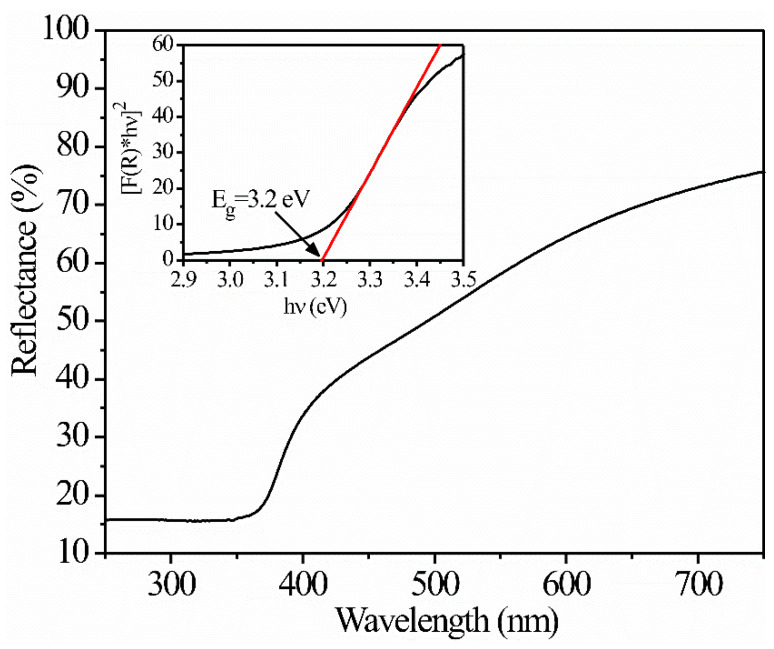
Reflection spectrum and relation F(R)*hv vs. wavelength (inset) of the ZnAl_2_O_4_.

The percentage of reflection and the forbidden bandwidth were determined from UV-vis spectra. Figure 4 shows the UV-vis reflectance spectrum of ZnAl_2_O_4_, where an increase in the percentage of reflection from 400 to 700 nm towards infrared and a strong absorption from 400 nm towards ultraviolet can be observed. To determine the value of the forbidden bandwidth, the Kubelka–Munk (K–M) theory was considered in which the absorption coefficient is proportional to the reflectance [31]. Hence, from Tauc’s formula, $\alpha \left(hv\right)\approx B{\left(hv-E_{g}\right)}^{n}$, and if $\alpha \approx F\left(R\right)=\frac{{\left(1-R\right)}^{2}}{2R}$, where *R* is the reflectance and *n* = 1/2 for a direct transition, the forbidden bandwidth’s value *E_g_* for the spinel ZnAl_2_O_4_ was obtained from the graph of ${\left(F\left(R\right)hv\right)}^{2}$ versus hv. As can be seen in Figure 4 (see inset), the value was 3.2 eV, which is less than the theoretically predicted value but similar to that experimentally reported [32,33].

### 3.3. SEM Analysis

**Figure 5:**
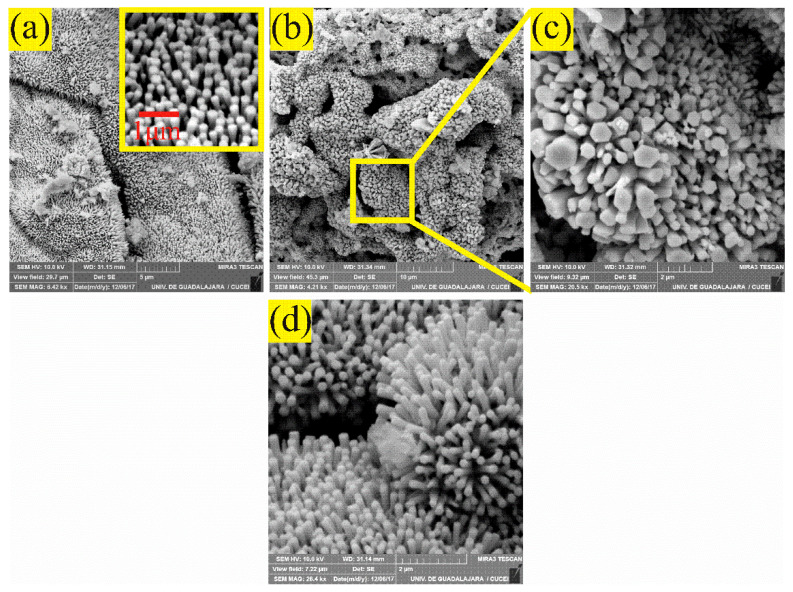
SEM images showing the formation of microrods at 300 °C, at magnifications: (**a**) 6.42 kX, (**b**) 4.21 kX, (**c**) 20.5 kX, and (**d**) 26.4 kX.

Figure 5 shows SEM images of the ZnAl_2_O_4_ obtained by calcination at 300 °C. For the analysis of the material’s microstructure, four different magnifications were employed: (a) 6.42 kX, (b) 4.21 kX, (c) 20.5 kX, and (d) 26.4 kX.

**Figure 6:**
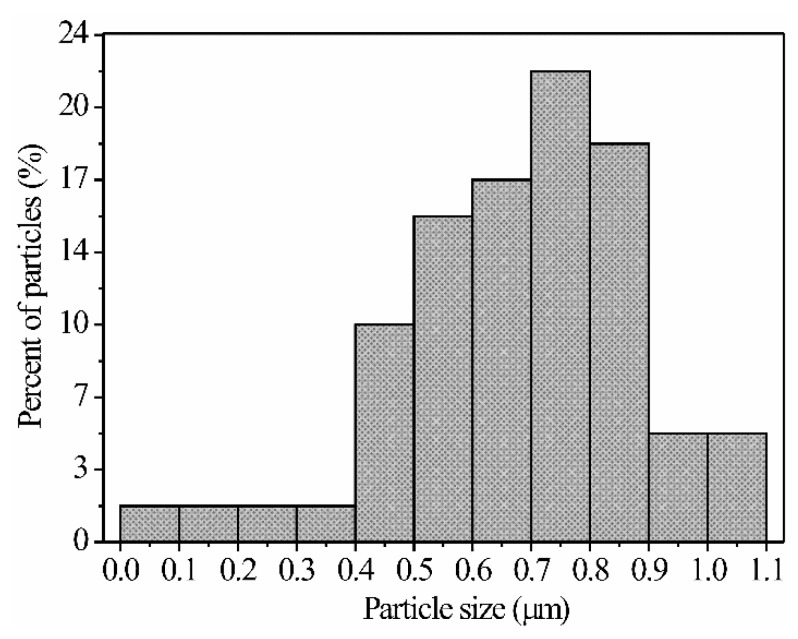
Size distribution of the ZnAl_2_O_4_ microrods obtained at 300 °C.

These photomicrographs show the multidirectional growth of a large number of microrods, which are distributed throughout the material’s surface (similar to a tapestry, see Figure 5a,b). These microrods are positioned next to each other, almost in an orderly manner, causing a coated surface of the microstructure, as shown in the inset of Figure 5a. It can also be observed that the microrods grow from areas located on microplates that act as a substrate. Such microrods agglomerate in a way that they tend to form a sphere-like geometry (see Figure 5b,c). Analyzing several SEM images, the length of the microrods was estimated in the range of 0.048 to 1.05 µm, with an average size of ~0.678 µm and a standard deviation of ~0.20 µm (see Figure 6). Observing another area of material’s surface, it is corroborated that the compound’s microstructure is formed by microrods that grow due to the effect of the temperature and the ethylenediamine concentration used during the synthesis process. The diameter of the microrods was estimated at ~0.398 µm on average. In general, it is observed that the surface of the ZnAl_2_O_4_ shows the same morphology, which can be seen in Figure 5d. In particular, the use of ethylenediamine as a way to produce 1D structures, such as rods, tubes, and wires, has been previously reported in the literature [34,35]. In our case, ethylenediamine acts as a template that is first incorporated into the inorganic network to later escape from it forming morphologies, such as the ones shown in Figure 5. Therefore, the presence of ethylenediamine determines the geometric features of the formed nuclei, which grow during the heat treatments [5]. We also have reported in previous works that by using ethylenediamine during the synthesis process, it is possible to obtain different types of morphologies [5,36,37,38], like microrods, nanorods, microspheres, microoctahedra, and nanoparticles, which have been successfully proved as potential gas sensors for the detection of CO, CO_2_, and C_3_H_8_ atmospheres [39]. Additionally, other authors, such as Michel et al. [40], used ethylenediamine for the synthesis of the semiconductor oxide CoSb_2_O_6_ in order to obtain nanoparticles to be applied in the detection of CO_2_ and O_2_. In general, the microstructure shown in Figure 5 follows the crystallization principles described by LaMer and Dinegar [41].

### 3.4. TEM Analysis

**Figure 7:**
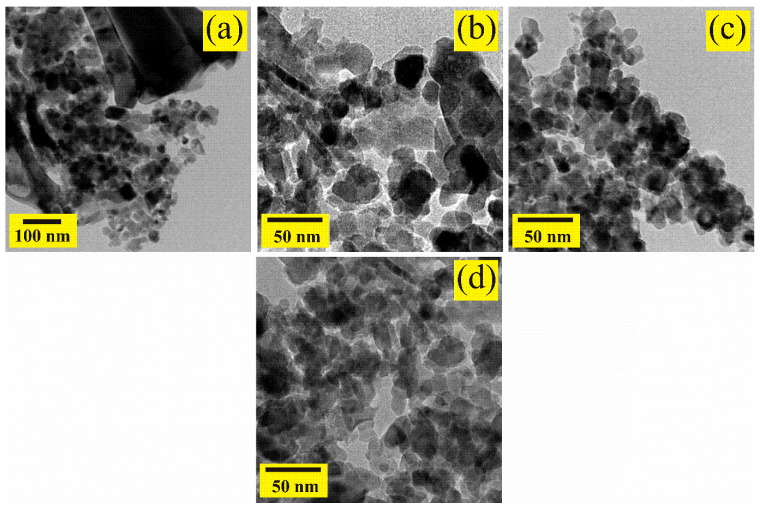
TEM images showing the individual characteristics of the ZnAl_2_O_4_ nanoparticles calcined at 300 °C: (**a**) growth of rods and nanoparticles, (**b**) agglomeration of nanoparticles to form the rods, (**c**) agglomeration of individual nanoparticles, (**d**) nucleation of different nanoparticle morphologies.

For a more detailed analysis of the individual particles that made up the ZnAl_2_O_4_ oxide, transmission electron microscopy (TEM) was employed. Figure 7 show typical bright-field TEM images of the oxide calcined at 300 °C.

**Figure 8:**
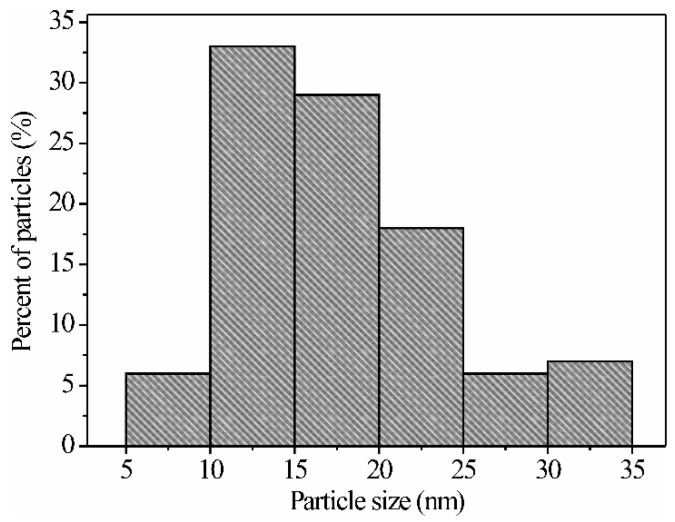
Nanoparticle size distribution for the ZnAl_2_O_4_ calcined at 300 °C.

In the TEM images, dark areas are observed that are attributed to the poor transmission of electrons caused by the agglomeration of nanoparticles on the material’s surface. It is mentioned in the literature that, when using ethylenediamine during the synthesis process, particle agglomeration occurs, where the thermal treatment (in our case at 300 °C) plays a key role in obtaining agglomerated areas of nanoparticles, as shown in Figure 7a–d [37,39]. In a more detailed study of the TEM images, we corroborated our SEM analysis where the rod growth and nucleation are observed, as well as other nanoparticles with no apparent shape (Figure 7a). Furthermore, it can be seen that the nanoparticles agglomerated one with the other until the nanorod morphology shown in Figure 7b was obtained. Regarding Figure 7c,d, it is observed that the surface of the ZnAl_2_O_4_ is made up of irregularly shaped nanometric-sized particles, which are joined by coalescence. The visible dispersion of the nanoparticles is attributed to the preparation technique for the TEM analysis, which required that the ZnAl_2_O_4_ was dispersed by ultrasound. The particle size, in a range from 5 to 35 nm (with an average of ~18.2 nm and a standard deviation of ~6.5 nm), was estimated by examining several TEM images (Figure 8).

**Figure 9:**
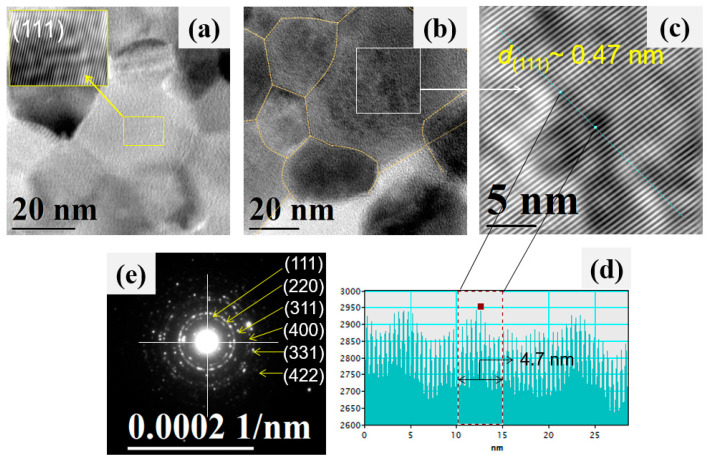
(**a**,**b**) TEM images showing the grain boundaries of the ZnAl_2_O_4_ nanoparticles, (**c**) HRTEM image where the oxide’s crystalline planes can be clearly identified, (**d**) histogram to estimate the crystals’ interplanar spacing, (**e**) electron diffraction pattern (SAED) from a selected area on the surface of a nanoparticle.

Figure 9 shows high-resolution TEM images (HRTEM) of the ZnAl_2_O_4_ nanoparticles obtained at 300 °C, a typical electron diffraction pattern of the oxide, and a histogram that corroborates the interplanar spacing of the ZnAl_2_O_4_. In Figure 9a,b, it can be seen that the material’s surface consisted of very fine and irregular nanoparticles (~20 nm in size). The borders between the individual nanoparticles were drawn with yellow-dotted lines. We could corroborate in these individual nanoparticles the presence of the oxide’s crystalline planes. Selecting some areas of the particle surfaces, we could estimate the distance between the planes at ~0.47 nm, which corresponded to the crystalline plane (111) of the spinel’s crystal structure, as shown in Figure 9a–d. Figure 9e shows a typical electron-diffraction pattern of the ZnAl_2_O_4_ nanoparticles, where the characteristic reflection rings of a nanometric polycrystalline material are observed. Accordingly, the first six reflections corresponding to the crystalline planes (111), (220), (311), (400), (331), and (422) were indexed, confirming the sample’s local crystallinity, consistently with the XRD study (see Figure 2).

### 3.5. Gas Sensing Tests

**Figure 10:**
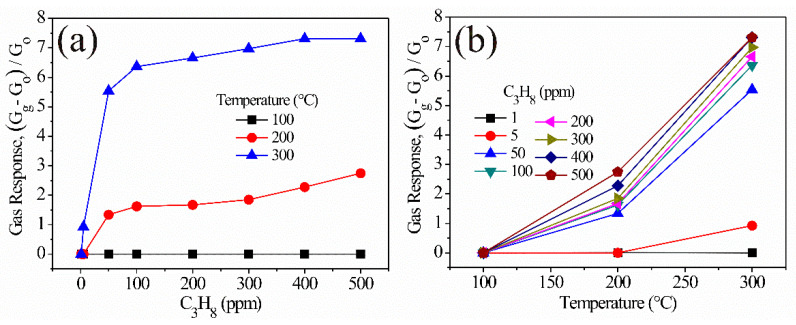
Response of ZnAl_2_O_4_ pellets as a function of (**a**) propane concentration, (**b**) operating temperature.

The results of the static detection tests on the ZnAl_2_O_4_ pellets at different operating temperatures and propane concentrations are shown in Figure 10. The propane concentrations were 1, 5, 50, 100, 200, 300, 400, and 500 ppm, while the temperatures were 100, 200, and 300 °C. As can be seen in Figure 10, the ZnAl_2_O_4_ shows an increase in its response as the propane concentration and the working temperature increase during the test. However, at 100 °C, the pellets did not show changes in electrical resistance with increasing concentration and operating temperature (see Figure 10a,b). This behavior is attributed to the fact that the relatively low-temperature is not enough for the reaction of the pellets’ surface and the test gas. This caused that at 100 °C, oxygen desorption did not occur [38,39] and, therefore, a low response of the material. Some authors also observed the poor response that shows some semiconductors at low temperatures (in our case at 100 °C), such as ours. They attributed such phenomenon to the fact that the oxygen species present (usually $O_{2}^{-}$ [2]) do not react with the test gas nor with the pellets’ surface (that is, adsorption and desorption processes do not occur) [5,10,12,17], provoking the absence of changes in the material’s electrical resistance [5,36] (see Figure 10). On the contrary, when increasing the operating temperature to 200 and 300 °C, a very significant increase in the response of the pellets was recorded (see Figure 10a,b). The increases in response are largely due to the fact that, with increasing thermal energy, $O^{-}$ oxygen species are easily adsorbed on the surface of the pellets [2,42]. These species are more reactive and capable of causing a greater speed in the mobility of charge carriers, having as a consequence an improvement in sensitivity, which in turn promotes an increase in the response of the ZnAl_2_O_4_. Therefore, by increasing the temperature to 300 °C, the response increased 2.7 times more than that measured at 200 °C (2.8 at 200 °C and 7.8 at 300 °C, both at 500 ppm of propane). The response increase is associated with the increase in the concentration of the propane gas that reacts with chemisorbed oxygen (ionic oxygen) on the material’s surface due to the rise of temperature. That caused variations in the semiconductor’s electrical resistance (or conductivity) and the increase of the sensor’s response [5,14,15,36]. The trend shown in Figure 10 is consistent with the results of similar oxides reported in the literature [2,5,36,43].

As seen in Figure 10a, at a propane concentration of 50 ppm, and at 200 and 300 °C, the response increased significantly. However, when the concentration was increased to 200, 300, 400, and 500 ppm, no significant changes were observed. Even at 300 °C, there was no change in the response from 400 to 500 ppm. This could be explained by the saturation of the sensor’s surface. Desorption and adsorption of gas molecules were in equilibrium at these concentrations, which meant that additional molecules could not be adsorbed directly at the sensor’s surface, causing a delayed contribution to resistance changes.

**Figure 11:**
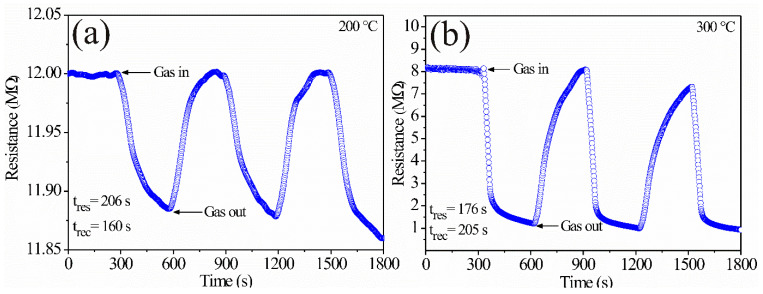
Dynamic response of ZnAl_2_O_4_ pellets in an air–propane mixture at temperatures: (**a**) 200 °C, (**b**) 300 °C.

**Figure 12:**
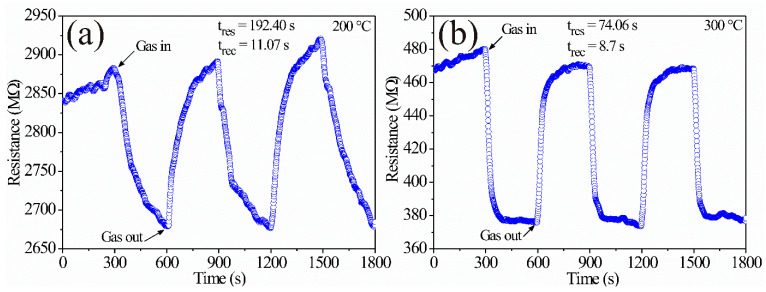
Dynamic response of ZnAl_2_O_4_ thick films in air–propane atmospheres at temperatures: (**a**) 200 °C, (**b**) 300 °C.

Dynamic tests on pellets and thick films made from ZnAl_2_O_4_ powders were carried out alternatively in air–propane atmospheres by the two-tips method using a direct current signal (DC). The variation of the electrical resistance was monitored while injecting synthetic air (20% O_2_, 80% N_2_) as a stabilizing gas for the pellets (500 mL/min of synth. air) and the films (1500 mL/min of synth. air). Propane’s concentration was 1000 ppm in balance with N_2_ (air–propane) at 200 and 300 °C in both cases. Material’s dynamic response was measured as follows: first, the pellets and the thick films were stabilized in synthetic air for 5 min at each temperature; subsequently, propane was injected for 5 min, provoking changes in the electrical resistance of the pellets and the thick films; then, the propane was withdrawn leaving the pellets and the thick films stabilizing in synthetic air for 5 min up to reaching the initial values of the electrical resistance. This process was repeated cyclically for each temperature (200 and 300 °C). Figure 11 and Figure 12 show the change in electrical resistance as a function of time at the given temperatures and propane concentration. As can be observed, the cycle repetitions are indicative of good reproducibility. In addition, as expected, in both cases, the oxide powders showed a decrease in electrical resistance (or an increase in conductivity) when the propane was introduced into the measurement chamber. The change was more noticeable at 300 °C. According to Figure 11a,b, the variations in electrical resistance were estimated at 0.126 MΩ (on average) at 200 °C, while at 300 °C it was calculated at 6.677 MΩ. The response and recovery times were calculated according to reference [44], which considered the changes in electrical resistance as a function of time. As in reference [45], we considered 90% of the total response and 10% of the saturation values in air. Therefore, when applying these criteria to the results shown in Figure 11, we estimated the response and recovery times at 200 °C were 206 and 160 s. At 300 °C, response and recovery times were 176 s and 205 s.

Similar trends were obtained on the thick films. The results are depicted in Figure 12a,b, where changes in electrical resistance are observed as a function of time at different operating temperatures. It can be verified that the effect of temperature and propane concentration played an important role during the tests. As in the pellets’ case, the changes in the electrical resistance fluctuated due to the reaction that occurred between propane and oxygen on the thick film’s surface at the given operating temperature, causing a decrease in electric resistance as a function of time. The average variation of the electrical resistance at 200 °C was 174.03 kΩ, with a response time of 192.40 s and a recovery time of 11.07 s, while at 300 °C it was 96.05 kΩ, with a response time of 74.06 s and a recovery time of 8.7 s. Again, as in the case of the dynamic response on the pellets, we considered references [44,45] to calculate the response and recovery times.

The response depicted in Figure 11 and Figure 12 is commonly observed in type-n semiconductor oxides [33] when they are exposed to atmospheres similar to ours [14,43]. Again, we consider that the increase in operating temperature (in our case from 200 to 300 °C) was a determining factor in the pellet’s response due to the presence of oxygen during the tests. References [2,46] report that several oxygen species appear as a consequence of the temperature during gas detection experiments: when testing at temperatures below 150 °C, the predominant oxygen species is $O_{2}^{-}$, while at temperatures greater than 150 °C, the predominant oxygen species are $O^{-}$ and $O^{2-}$, which are more reactive than $O_{2}^{-}$ [5,36,46]; oxygen reactions produced at temperatures above 150 °C (in our case 200 and 300 °C) cause an increase in the reaction kinetics of the gas molecules on the pellet surfaces and thereby a sensitivity increase. It has been reported that other factors that affect the test gas chemisorption are both the presence of oxygen and the material’s microstructure (size and morphology) [47] since if the particle size is less than 2 times the thickness of the outer layer (L_S_), the adsorbed oxygen species are those that lead to a greater material’s response [43,48], as occurred in our case.

In the case of the pellets, the sensor’s resistance varied from ~8 MΩ without gas to ~1 MΩ in propane at 300 °C (Figure 11b); the ratio R_0_/R_G_ was 8. Instead, for the thick films, at the same temperature and propane concentration, the change in the resistance varied from ~480 MΩ to ~380 MΩ (Figure 12b); the ratio R_0_/R_G_ was 1.26. The increased pellet response could be attributed to the preparation of the sensor: pressure was applied to the powder, which should decrease the average distance between the particles. The current between the grains must be strongly controlled by the Schottky barriers between the grains of the material. On the other hand, the thick films were drip prepared. As a consequence, their particles had, on average, a greater distance (i.e., there was less contact) between them. Therefore, higher electrical resistance was generated with the associated tunneling effect. Another effect of the grain compaction was observed in the response times: there was greater gas mobility through the film (associated with more pores) compared to the pellets.

In general, the response of a semiconductor material (such as the one studied in this work) is based on the mechanism that involves changes in the conductance (or electrical resistance) produced by the electron transfer that occurs during chemical adsorption [8,14,43]. Depending on the semiconductor type (p or n), the surface charge carriers may increase or decrease, as appropriate [14]. The mobility and variations of the charge carriers depend on the temperature [49], the morphology, and the particle size of the semiconductor (which in our case was ~18.2 nm) [47,49,50]. This means that the interaction between the oxygen and the semiconductor’s surface will be more active due to the effect of temperature. That will provoke more significant changes in the semiconductor’s electrical resistance, therefore, meaning a greater response, as happened in this work.

The sensitivity of the ZnAl_2_O_4_ prepared in this work was compared with previous studies on propane gas sensing. For example, thin-films of SnO_2_ showed a sensitivity of 0.7 at a propane concentration of 300 ppm at a temperature of 300 °C [51]. At the same experimental conditions, thin-films of ZnO exhibited a response of 0.4 [52]. The trirutile-type ZnSb_2_O_6_ [53] and CoSb_2_O_6_ [54] showed responses of 1.3 and 4.8, respectively, to 300 ppm of propane at temperatures between 250 and 300 °C. The cited studies do not report information on response and recovery times. The results obtained in the present study highlight the synthesized spinel as a promising material in gas sensing applications.

## 4. Conclusions

In this work, ZnAl_2_O_4_ nanoparticles were synthesized by the microwave-assisted colloidal method, which is a simple and economical process that does not require sophisticated equipment for obtaining both nanometric particle sizes and the crystalline phase at low temperatures. The powders were studied chemically by XPS, which provided information on the oxidation states of the Zn and the Al. The bandgap of the oxide was determined by UV-vis, obtaining a value of 3.2 eV. The oxide nanoparticles showed an increase in sensitivity as the propane concentration and the operating temperature were also raised. The maximum sensitivity value in the static test was ~7.8 for a propane concentration of 500 ppm at 300 °C. The dynamic tests on pellets and thick films of ZnAl_2_O_4_ revealed that with the increase in operating temperature and test gas concentration, the response of the nanoparticles improved substantially, which was reflected in the changes in their electrical resistance. The best response was at a temperature of 300 °C with short response and recovery times. According to our results, ZnAl_2_O_4_ nanoparticles can be a viable option to be used as a propane sensor.

## Data Availability

The data that support the findings of this study are available from the corresponding authors upon request.

## References

[1] Rounaghi S.A., Vanpoucke D.E.P., Esmaeili E., Scudino S., Eckert J. (2019). Synthesis, characterization and thermodynamic stability of nanostructured ε-iron carbonitride powder prepared by a solid-state mechanochemical route. J. Alloys Compd..

[2] Wetchakun K., Samerjai T., Tamaekong N., Liewhiran C., Siriwong C., Kruefu V., Wisitsoraat A., Tuantranont A., Phanichphant A. (2011). Semiconducting metal oxides as sensors for environmentally hazardous gases. Sens. Actuators B Chem..

[3] Xavier-Stango S.A., Vijayalakshmi U. (2019). Synthesis and characterization of hydroxyapatite/carboxylic acid functionalized MWCNTS composites and its triple layer coatings for biomedical applications. Ceram. Int..

[4] Korotcenkov G., Cho B.K. (2017). Metal oxide composites in conductometric gas sensors: Achievements and challenges. Sens. Actuators B Chem..

[5] Gildo-Ortiz L., Guillén-Bonilla H., Rodríguez-Betancourtt V.M., Blanco-Alonso O., Guillén-Bonilla A., Santoyo-Salazar J., X Romero-Ibarra V.M., Reyes-Gómez J. (2018). Key processing of porous and fibrous LaCoO_3_ nanostructures for successful CO and propane sensing. Ceram. Int..

[6] Roya J., Chandra S., Maitra S. (2019). Nanotechnology in castable refractory. Ceram. Int..

[7] Matijevic E. (1994). Uniform inorganic colloid dispersions. Achievements and challenges. Langmuir.

[8] Michel C.R., Guillén-Bonilla H., Martínez-Preciado A.H., Morán-Lázaro J.P. (2009). Synthesis and gas sensing properties of nanostructured CoSb_2_O_6_ microspheres. Sens. Actuators B Chem..

[9] Gao X., Zhang T. (2018). An overview: Facet-dependent metal oxide semiconductor gas sensors. Sens. Actuators B Chem..

[10] Degler D. (2018). Trends and Advances in the Characterization of Gas Sensing Materials Based on Semiconducting Oxides. Sensors.

[11] Fragoso-Mora J.R., Matatagui D., Bahos F.A., Fontecha J., Fernandez M.J., Santos J.P., Sayago I., Gràcia I., Horrillo M.C. (2018). Gas sensors based on elasticity changes of nanoparticle layers. Sens. Actuators B Chem..

[12] Yang X., Zhang S., Yu Q., Zhao L., Sun P., Wang T., Liu F., Yan X., Gao Y., Liang X. (2019). One step synthesis of branched SnO_2_/ZnO heterostructures and their enhanced gas-sensing properties. Sens. Actuators B Chem..

[13] Ding J.-C., Li H.-Y., Cao T.-C., Cai Z.-X., Wang X.-X., Guo X. (2017). Characteristics and sensing properties of CO gas sensors based on LaCo_1__−x_FexO_3_ nanoparticles. Solid State Ion..

[14] Singh S., Singh A., Singh A., Tandon P. (2020). A stable and highly sensitive room-temperature liquefied petroleum gas sensor based on nanocubes/cuboids of zinc antimonate. RSC Adv..

[15] Morán-Lázaro J.P., Blanco O., Rodríguez-Betancourtt V.-M., Reyes-Gómeze J., Michel C.R. (2016). Enhanced CO_2_-sensing response of nanostructured cobalt aluminate synthesized using a microwave-assisted colloidal method. Sens. Actuators B Chem..

[16] Balamurugana C., Maheswari A.R., Lee D.-W. (2014). Structural, optical, and selective ethanol sensing properties of p-type semiconducting CoNb_2_O_6_ nanopowder. Sens. Actuators B Chem..

[17] Cheng B., Ouyang Z., Tian B., Xiao Y., Lei S. (2013). Porous ZnAl_2_O_4_ spinel nanorods: High sensitivity humidity sensors. Ceram. Int..

[18] Fernandez-Osorio A., Rivera C.E., Vazquez-Olmos A., Chavez J. (2015). Luminescent ceramic nano-pigments based on terbium-doped zinc aluminate: Synthesis, properties and performance. Dyes Pigment..

[19] Wang S.-F., Tsai Y.-T., Chu J.P. (2016). Resistive switching characteristics of a spinel ZnAl_2_O_4_ thin film prepared by radio frequency sputtering. Ceram. Int..

[20] Dhak D., Pramanik P. (2006). Particle size comparison of soft-chemically prepared transition metal (Co, Ni, Cu, Zn) aluminate spinels. J. Am. Ceram. Soc..

[21] Ianoş R., Băbuţă R., Păcurariu C., Lazău R., Istratie R., Butaciu C. (2017). Combustion synthesis of ZnAl_2_O_4_ powders with tuned surface area. Ceram. Int..

[22] Tangcharoen T., Thienprasert J.-T., Kongmark C. (2018). Optical properties and versatile photocatalytic degradation ability of MAl_2_O_4_ (M = Ni, Cu, Zn) aluminate spinel nanoparticles. J. Mater. Sci. Mater. Electron..

[23] Cullity B.D. (1956). Elements of X-ray Diffraction.

[24] Ianos R., Borcanescu S., Lazau R. (2014). Large surface area ZnAl_2_O_4_ powders prepared by a modified combustion technique. Chem. Eng. J..

[25] Muñoz-Flores J., Herrera-Gomez A. (2012). Resolving overlapping peaks in ARXPS data: The effect of noise and fitting method. J. Electron Spectrosc. Relat. Phenom..

[26] Zhang D., Du C., Chen J., Shi Q., Wang Q., Li S., Wang W., Yan X., Fan Q. (2018). Improvement of structural and optical properties of ZnAl_2_O_4_:Cr^3+^ ceramics with surface modification by using various concentrations of zinc acetate. J. Sol-Gel Sci. Technol..

[27] Cabello G., Lillo L., Caro C., Seguel M., Sandoval C., Buono-Core G.E., Chornik B., Flores M. (2016). A photochemical proposal for the preparation of ZnAl_2_O_4_ and MgAl_2_O_4_ thin films from b-diketonate complex precursors. Mater. Res. Bull..

[28] Ceballos-Sanchez O., Sanchez-Martinez A., Vazquez-Lepe M.O., Duong T., Arroyave R., Espinosa-Magaña F., Herrera-Gómez A. (2012). Mass transport and thermal stability of TiN/Al_2_O_3_/InGaAs nanofilms. J. Appl. Phys..

[29] Alfaro-Cruz M.R., Ceballos-Sanchez O., Luévano-Hipólito E., Torres-Martínez L.M. (2018). ZnO thin films deposited by RF magnetron sputtering: Effects of the annealing and atmosphere conditions on the photocatalytic hydrogen production. Int. J. Hydrogen Energy.

[30] Iaiche S., Djelloul A. (2015). ZnO/ZnAl_2_O_4_ Nanocomposite films studied by X-ray diffraction, FTIR, and X-ray photoelectron spectroscopy. J. Spectrosc..

[31] López R., Gómez R. (2012). Band-gap energy estimation from diffuse reflectance measurements on sol–gel and commercial TiO_2_: A comparative study. J Sol-Gel Sci. Technol..

[32] Motloung S.V., Dejene F.B., Ntwaeaborwa O.M., Swart H.C. (2014). Effects of catalyst/zinc mole fraction on ZnAl_2_O_4_:0.01% Cr^3+^ nanocrystals synthesized using sol–gel process. Mater. Res. Express..

[33] Dixit H., Tandon N., Cottenier S., Saniz R., Lamoen D., Partoens B., Speybroeck V.V., Waroquier M. (2011). Electronic structure and band gap of zinc spinel oxides beyond LDA: ZnAl_2_O_4_, ZnGa_2_O_4_ and ZnIn_2_O_4_. New J. Phys..

[34] Wang X., Li Y. (2006). Solution-based synthetic strategies for 1-D nanostructures. Inorg. Chem..

[35] Deng Z.-X., Wang C., Sun X.-M., Li Y.D. (2002). Structure-directing coordination template effect of ethylenediamine in formations of ZnS and ZnSe nanocrystallites via solvothermal route. Inorg. Chem..

[36] Guillen-Bonilla H., Olvera-Amador M.L., Casallas-Moreno Y.L., Guillen-Bonilla J.T., Guillen-Bonilla A., Gildo-Ortiz L., Moran-Lazaro J.P., Santoyo-Salazar J., Rodriguez-Betancourtt V.-M. (2019). Synthesis and characterization of nickel antimonate nanoparticles: Sensing properties in propane and carbon monoxide. J. Mater. Sci. Mater. Electron..

[37] Guillen-Bonilla J.T., Guillen-Bonilla H., Rodríguez-Betancourtt V.M., Casillas-Zamora A., Ramírez-Ortega J.A., Gildo-Ortiz L., Sánchez-Morales M.E., Blanco-Alonso O., Guillén-Bonilla A. (2019). Carbone monoxide (CO) detection device based on the nickel antimonate oxide and a DC electronic circuit. Appl. Sci..

[38] Guillen-Bonilla H., Rodríguez-Betancourtt V.M., Guillen-Bonilla J.T., Gildo-Ortiz L., Guillén-Bonilla A., Casallas-Moreno Y.L., Blanco-Alonso O., Reyes-Gómez J. (2018). Sensitivity tests of pellets made from manganese antimonate nanoparticles in carbon monoxide and propane atmospheres. Sensors.

[39] Casillas-Zamora A., Guillen-Bonilla J.T., Guillén-Bonilla A., Rodríguez-Betancourtt M., Casallas-Moreno Y.L., Gildo-Ortiz L., Olvera-Amador M.L., Tomás S.A., Guillen-Bonilla H. (2020). Synthesis of MnSb_2_O_6_ powders through a simple low-temperature method and their test as a gas sensor. J. Mater. Sci. Mater. Electron..

[40] Michel C.R., Martínez-Preciado A.H., Morán-Lázaro J.P. (2009). Effect of the frequency on the gas sensing response of CoSb_2_O_6_ prepared by a colloidal method. Sens. Actuators B Chem..

[41] LaMer V.K., Dinegar R.H. (1950). Theory, production and mechanism of formation of monodispersed hydrosols. J. Am. Chem. Soc..

[42] Wang C., Yin L., Zhang L., Xiang D., Gao R. (2010). Metal oxide gas sensors: Sensitivity and influencing factors. Sensors.

[43] Singh S., Singh A., Singh A., Rathore S., Yadav B.C., Tandon P. (2020). Nanostructured cobalt antimonate: A fast responsive and highly stable sensing material for liquefied petroleum gas detection at room temperature. RSC Adv..

[44] Arshak K., Moore E., Lyons G.M., Harris J., Clifford S. (2004). A review of gas sensors employed in electronic nose applications. Sens. Rev..

[45] Cavallari M.R., Pastrana L.M., Sosa C.D.F., Marquina A.M.R., Izquierdo J.E.E., Fonseca F.J., Amorim C.A.d., Paterno L.G., Kymissis I. (2021). Organic thin-film transistors as gas sensors: A Review. Materials.

[46] Chang S.C. (1979). Oxygen chemisorption on tin oxide: Correlation between electrical conductivity and EPR measurements. J. Vac. Sci. Technol..

[47] Fioravanti A., Marani P., Morandi S., Lettieri S., Mazzocchi M., Sacerdoti M., Carotta M.C. (2021). Growth Mechanisms of ZnO Micro-Nanomorphologies and Their Role in Enhancing Gas Sensing Properties. Sensors.

[48] Lin T., Lv X., Li S., Wang Q. (2017). The morphologies of the semiconductor oxides and their gas-sensing properties. Sensors.

[49] Alrammouz R., Podlecki J., Abboud P., Sorli B., Habchi R. (2018). A review on flexible gas sensors: From materials to devices. Sens. Actuators A Phys..

[50] Zhou Q., Chen W., Xu L., Kumar R., Gui Y., Zhao Z., Tang C., Zhu S. (2018). Highly sensitive carbon monoxide (CO) gas sensors based on Ni and Zn doped SnO_2_ nanomaterials. Ceram. Int..

[51] Gómez-Pozos H., González-Vidal J.L., Torres G.A., Olvera M.L., Castañeda L. (2013). Physical characterization and effect of effective surface area on the sensing properties of tin dioxide thin solid films in a propane atmosphere. Sensors.

[52] Gómez H., González J.L., Torres G.A., Rodríguez J., Maldonado A., Olvera M.L., Acosta D.R., Avendaño M., Castañeda L. (2013). Chromium and ruthenium-doped zinc oxide thin films for propane sensing applications. Sensors.

[53] Guillén-Bonilla H., Rodríguez-Betancourtt V.M., Guillén Bonilla J.T., Reyes-Gómez J., Gildo-Ortiz L., Flores-Martínez M., Olvera-Amador M.L., Santoyo-Salazar J. (2015). CO and C_3_H_8_ sensitivity behavior of zinc antimonate prepared by a microwaveassisted solution method. J. Nanomater..

[54] Guillén-Bonilla H., Gildo-Ortiz V., Olvera-Amador M.L., Santoyo-Salazar J., Rodríguez-Betancourtt V.M., Guillén-Bonilla A., Reyes-Gómez J. (2015). Sensitivity of mesoporous CoSb_2_O_6_ nanoparticles to gaseous CO and C_3_H_8_ at low temperatures. J. Nanomater..

